# A novel multitask learning algorithm for tasks with distinct chemical space: zebrafish toxicity prediction as an example

**DOI:** 10.1186/s13321-024-00891-4

**Published:** 2024-08-02

**Authors:** Run-Hsin Lin, Pinpin Lin, Chia-Chi Wang, Chun-Wei Tung

**Affiliations:** 1 Institute of Biotechnology and Pharmaceutical Research, National Health Research Institutes, Miaoli County, 35053 Taiwan; 2 Graduate Institute of Data Science, College of Management, Taipei Medical University, Taipei, 10675 Taiwan; 3 National Institute of Environmental Health Sciences, National Health Research Institutes, Miaoli County, 35053 Taiwan; 4 Department and Graduate Institute of Veterinary Medicine, School of Veterinary Medicine, National Taiwan University, Taipei, 10617 Taiwan

**Keywords:** Chemical space, Developmental toxicity, Chemical toxicity, Multitask learning, Zebrafish

## Abstract

Data scarcity is one of the most critical issues impeding the development of prediction models for chemical effects. Multitask learning algorithms leveraging knowledge from relevant tasks showed potential for dealing with tasks with limited data. However, current multitask methods mainly focus on learning from datasets whose task labels are available for most of the training samples. Since datasets were generated for different purposes with distinct chemical spaces, the conventional multitask learning methods may not be suitable. This study presents a novel multitask learning method MTForestNet that can deal with data scarcity problems and learn from tasks with distinct chemical space. The MTForestNet consists of nodes of random forest classifiers organized in the form of a progressive network, where each node represents a random forest model learned from a specific task. To demonstrate the effectiveness of the MTForestNet, 48 zebrafish toxicity datasets were collected and utilized as an example. Among them, two tasks are very different from other tasks with only 1.3% common chemicals shared with other tasks. In an independent test, MTForestNet with a high area under the receiver operating characteristic curve (AUC) value of 0.911 provided superior performance over compared single-task and multitask methods. The overall toxicity derived from the developed models of zebrafish toxicity is well correlated with the experimentally determined overall toxicity. In addition, the outputs from the developed models of zebrafish toxicity can be utilized as features to boost the prediction of developmental toxicity. The developed models are effective for predicting zebrafish toxicity and the proposed MTForestNet is expected to be useful for tasks with distinct chemical space that can be applied in other tasks.

**Scieific contribution**

A novel multitask learning algorithm MTForestNet was proposed to address the challenges of developing models using datasets with distinct chemical space that is a common issue of cheminformatics tasks. As an example, zebrafish toxicity prediction models were developed using the proposed MTForestNet which provide superior performance over conventional single-task and multitask learning methods. In addition, the developed zebrafish toxicity prediction models can reduce animal testing.

**Supplementary Information:**

The online version contains supplementary material available at 10.1186/s13321-024-00891-4.

## Introduction

The development of prediction models for chemical bioactivity and toxicity using small data faces great challenges. While machine learning algorithms have been successfully applied to develop prediction models for various endpoints, the use of incomprehensive training data can lead to issues of limited applicability and overfitting. In contrast to the traditional single-task methods that learn a task at a time, multitask learning algorithms simultaneously learning multiple tasks are more relevant to the human learning process for leveraging cross-domain knowledge. Data from relevant learning tasks can augment the learning and avoid overfitting issues. Shared knowledge learned from multitasks can improve model generalization and performance [1, 2].

Several cheminformatics studies have developed multitask learning approaches to improve model performance. Since multitask learning using neural networks with shared layers is intuitive, most of the cheminformatics studies used deep neural network-based implementation [3]. For example, a multitask model based on graph convolutional neural networks (GCN) can deal with data imbalance issues for Tox21 data (Li et al. 2023). GCN was also found to be useful for predicting bioactivity data for tasks of several hundred kinases [4]. Datasets of Ames test results for five strains were simultaneously learned using a multitask deep neural network with superior performance compared to single-task models [5]. In addition to the mechanism of shared layers, Siamese networks were proposed for learning shared parameters of neural networks to facilitate data-poor learning tasks [6]. In contrast to the neural network methods requiring extensive hyperparameter tuning, an ExtraTree-based multitask method has been implemented for predicting skin sensitizers [7].

While successful applications have been shown, current multitask applications in this field mainly focus on learning from datasets with many shared training samples among the tasks. For example, each of the extensively studied datasets of QM9, ClinTox, Sider and Tox21 in the previous multitask learning studies contains a set of chemicals with multiple labels [8–13]. For multitask learning based on multiple data sources, the chemical spaces of datasets are usually distinct. The applicability of the abovementioned multitask learning algorithms for tasks with distinct chemical space is largely unknown. In addition, hyperparameter tuning for multitask deep neural networks could be tedious and complicated [14]. The performance of conventional multitask deep neural networks varies in different applications [8]. In this regard, the development and assessment of multitask learning algorithms for tasks with distinct chemical spaces are therefore desirable.

This study took zebrafish toxicity prediction as an example. Zebrafish (Danio rerio) as an important model organism has been widely used for phenotype-based drug discovery and toxicity screening [15, 16]. A few studies have conducted large-scale toxicity screening for several thousands of chemicals based on zebrafish [17–22]. However, the application of the experimental methods for a large number of chemicals is still time- and resource-consuming. The development of prediction models for zebrafish toxicity based on available experimental data can be useful for prioritizing chemicals of concern for further experimental validation. Multitask learning is expected to further improve the prediction performance leveraging all available datasets. However, among the data sources [17–22], one data source represents a distinct chemical space sharing only 1.3% common chemicals with other data sources. The development of prediction models for zebrafish toxicity could be therefore challenging.

In this study, a dataset of chemical toxicity for zebrafish consisting of 48 toxicity endpoints was compiled from multiple data sources and divided into training, validation, and test datasets for model training, parameter tuning, and independent testing, respectively. A novel multitask learning algorithm named MTForestNet using random forest [23] as base learners with a stacking mechanism was developed to progressively improve the prediction performance of all learning tasks. The stacking mechanism is to iteratively train new models by using a concatenated feature vector consisting of original features with outputs from task-specific models of the previous layer. In terms of area under the receiver operating characteristic curve (AUC) value, the proposed algorithm achieved a high independent test performance of 0.911 representing a 26.3% improvement over the conventional single-task learning models. Also, the MTForestNet showed superior performance over conventional multitask learning methods. The proposed multitask learning method MTForestNet is expected to be useful for tasks with distinct chemical space and the developed model can be applied to the screening of chemicals with potential toxicity concerns for further experimental validation.

## Materials and method

### Data preprocessing

To develop multitask learning models for zebrafish toxicity, a total of four datasets consisting of 48 toxicity endpoints and 6,885 chemicals were collected from 6 experimental studies of zebrafish and zebrafish embryo toxicity [17–22]. The four tasks were named larva, embryo development, lipid metabolism, and embryo morphology. The tasks represent diverse endpoints of mortality, morphology, behavior, and development. This study focused on binary classification, therefore toxicity thresholds adopted from the reference studies were utilized to classify chemicals as toxic compounds. The other chemicals not fit the threshold are considered nontoxic compounds. Duplicates, mixtures, and chemicals without explicit structure information were excluded from the following analysis. Summary and details of the four datasets and 48 tasks are shown in Table 1 and Table S1, respectively. Among the tasks, 15 tasks are class imbalanced with less than or equal to 10% active/inactive ratio. In contrast, the task of developmental toxicity score (TOX_SCO) is with a high active/inactive ratio of 74.0%. Each chemical was then converted to a 1024-bit feature vector using the extended connectivity fingerprints of diameter 6 (ECFP). After the preprocessing, a total of 4854 non-duplicate chemicals and 48 tasks were included in the final datasets.

**Table 1:** Summary of the datasets used in this study

Dataset	No. of chemicals (initial/ processed dataset)	Endpoints	Toxicity threshold
Larva [20]	1060/1040	BMD10 (benchmark dose for 10% effect) for (1) 2 mortality endpoints (24 hpf and 120 hpf) (2) 3 morphology endpoints (24 hpf) (3) 17 morphology endpoints (120 hpf) (4) 2 behavioral endpoints (120 hpf)	BMD10 ≤ 1 mM
Embryo development [22]	1060/1040	LEL (Lowest Effect Level) for (1) 1 mortality endpoint (120 hpf) (2) 17 morphology endpoints (120 hpf)	LEL ≤ 64 uM
Lipid metabolism [18]	3806/3801	Overnight larva lipid metabolism inhibition (1) MIC (minimum inhibition concentration) (2) Score	(1) MIC ≤ 100 uM (1) score > 0
Embryo morphology [17, 19, 21]	959/934	LEL (Lowest Effect Level) for (1) The lowest LEL value of 18 cognition and behavior endpoints (120 hpf) (2) The lowest LEL of 17 sublethal endpoints (120 hpf) (3) Mortality (120 hpf)	LEL ≤ 64 uM
		Development toxicity score (120 hpf)	Score > 2.24

**Fig. 1:**
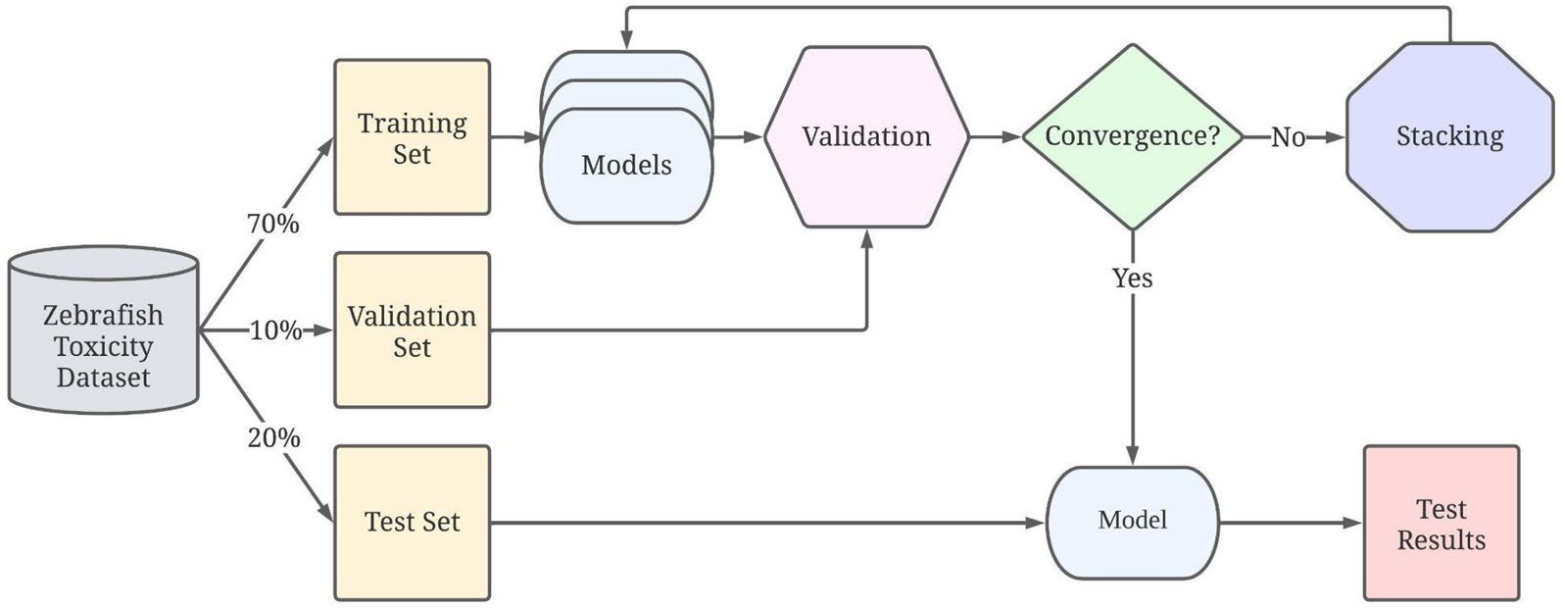
System flow of the proposed method

### MTForestNet

**Fig. 2:**
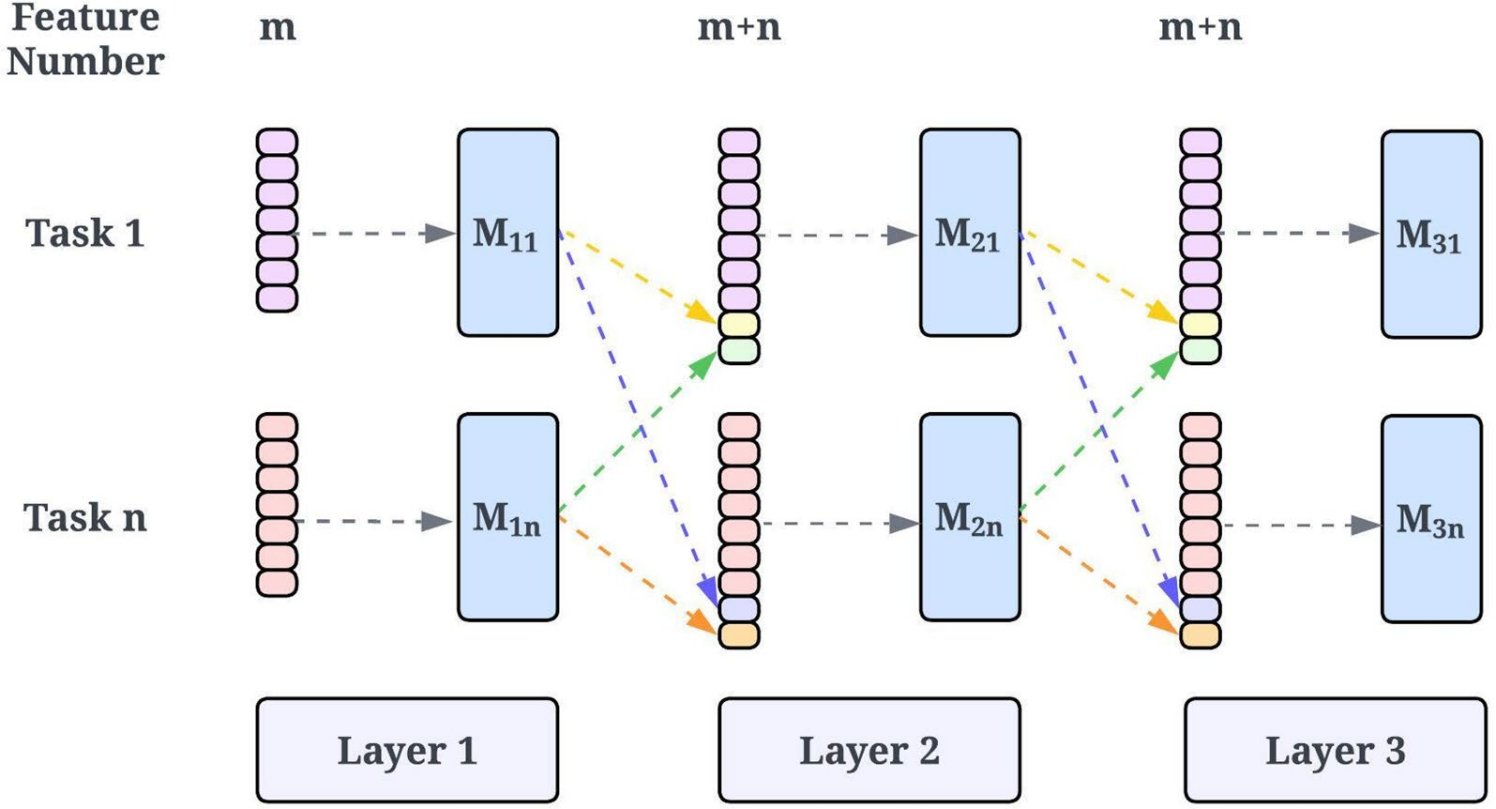
Schematic diagram of MTForestNet

As shown in Table 1, the datasets can be very different with distinct chemical spaces. To enable multitask learning for datasets with distinct chemical spaces, a progressive multitask learning strategy (MTForestNet) concatenating chemical fingerprint features and outputs of individual classifiers from the previous layer for accuracy improvement was proposed.  The system flow of the proposed method is shown in Fig. 1. The architecture of the MTForestNet is shown in Fig. 2. The classifier implemented in this study is the random forest [23] which can provide robust prediction performance in many studies [24–29]. In this study, the implementation of random forest was based on scikit-learn [30].

The training process started with the model training for each of the 48 learning tasks using random forest. The parameters of the number of trees (n_estimators), number of features (max_features) for building a tree, and random seed (random_state) for random forest training were set to 500, log2(feature number), and 8, respectively. To avoid overfitting issues and provide a reasonable estimation of prediction performance, each of the datasets was randomly split into a training set (70%), a validation set (10%), and a test set (20%). The training set was utilized for training models, while the validation set was utilized for parameter tuning. The remaining test set not involved in model training and tuning was utilized for estimating the prediction performance for unseen samples. The above-mentioned first step constitutes the first layer of MTForestNet and its prediction performance is considered the baseline of the traditional single-task learning approach where no information is shared between tasks.

To leverage the information learned from other learning tasks for model improvement, the original feature vectors (m = 1024) were concatenated with 48 score outputs (n = 48) predicted from the models of the first layer and utilized for training 48 models constituting the second layer. The validation set was utilized for calculating the prediction performance of the area under the receiver operating characteristic curve (AUC) of 48 models for each layer. The average AUC of the 48 models was taken as the overall performance for each layer. The procedure was conducted iteratively for building subsequent layers until no improvement in average AUC on the validation set was observed.

### Overall toxicity measurement for Zebrafish

Embryonic zebrafish metric (EZ Metric) is an overall measure of morbidity and mortality in embryonic zebrafish that has been utilized to prioritize the toxicity of diverse nanomaterials [31–33]. The metric is a weighted sum of 4 endpoints at 24 hpf (hours post fertilization) for mortality, delayed development, absence of spontaneous movement, and notochord malformation, and 17 endpoints at 120 hpf for mortality, organ malformation, and dysfunction. The weights defined in the previous study [33] and endpoints are shown in Table S3.

### Developmental toxicity prediction

To assess the performance improvement by incorporating the predicted features obtained from the developed multitask model, two published developmental toxicity datasets of DART and PDT representing developmental and reproductive toxicity [34] and prenatal developmental toxicity [35] in humans, respectively, were utilized for performance comparison. The DART dataset consists of 201 toxic and 91 nontoxic chemicals, while the PDT dataset comprises 660 toxic and 584 nontoxic chemicals. To avoid sampling bias, 100 runs of experiments were conducted in this study. For each run, the two datasets were randomly split into 80% training and 20% test sets.

The 48 binary prediction results were appended to the original 1024-bit ECFP feature vector to form a new 1072-bit feature vector. For each of the two datasets, two random forest-based models were trained using the corresponding training set and the 1024-bit and 1072-bit feature vectors. The parameters of the number of trees (n_estimators), number of features (max_features) for building a tree, and random seed (random_state) were set to 500, log2 (feature number), and 8, respectively.

Since the features of 48 zebrafish tasks can be redundant, the identification of task-relevant features can potentially improve the prediction performance. We implemented a sequential backward feature selection algorithm with conditional feature inclusion using the MLxtend package [36]. The feature set giving the highest area under the curve (AUC) value in tenfold cross-validation (10-CV) on the respective training set was selected for building the final model for the independent test. The average AUCs of the 100 runs were then calculated to represent the model performance.

## Results and discussion

### Distinct chemical space of the Zebrafish datasets

The chemical space of the four datasets collected from different sources was analyzed to characterize the learning problem. Two measurements of the highest percentage of common chemicals and average similarity were calculated for each pair of the four datasets. Since one dataset contains multiple subsets of endpoints, the percentage of common chemicals was calculated in a subset-wise manner. Given *n* subsets in dataset A and *m* subsets in dataset B, *n* × *m* percentage values of common chemicals were obtained by dividing the number of common chemicals by the number of unique chemicals in both subsets. The highest percentage value from subset-wise comparison was taken as the percentage of common chemicals between the two datasets. For calculating the average similarity, an average of Tanimoto similarities for *n* × *m* subset pairs was utilized to represent the overall similarity between the two datasets.

**Fig. 3:**
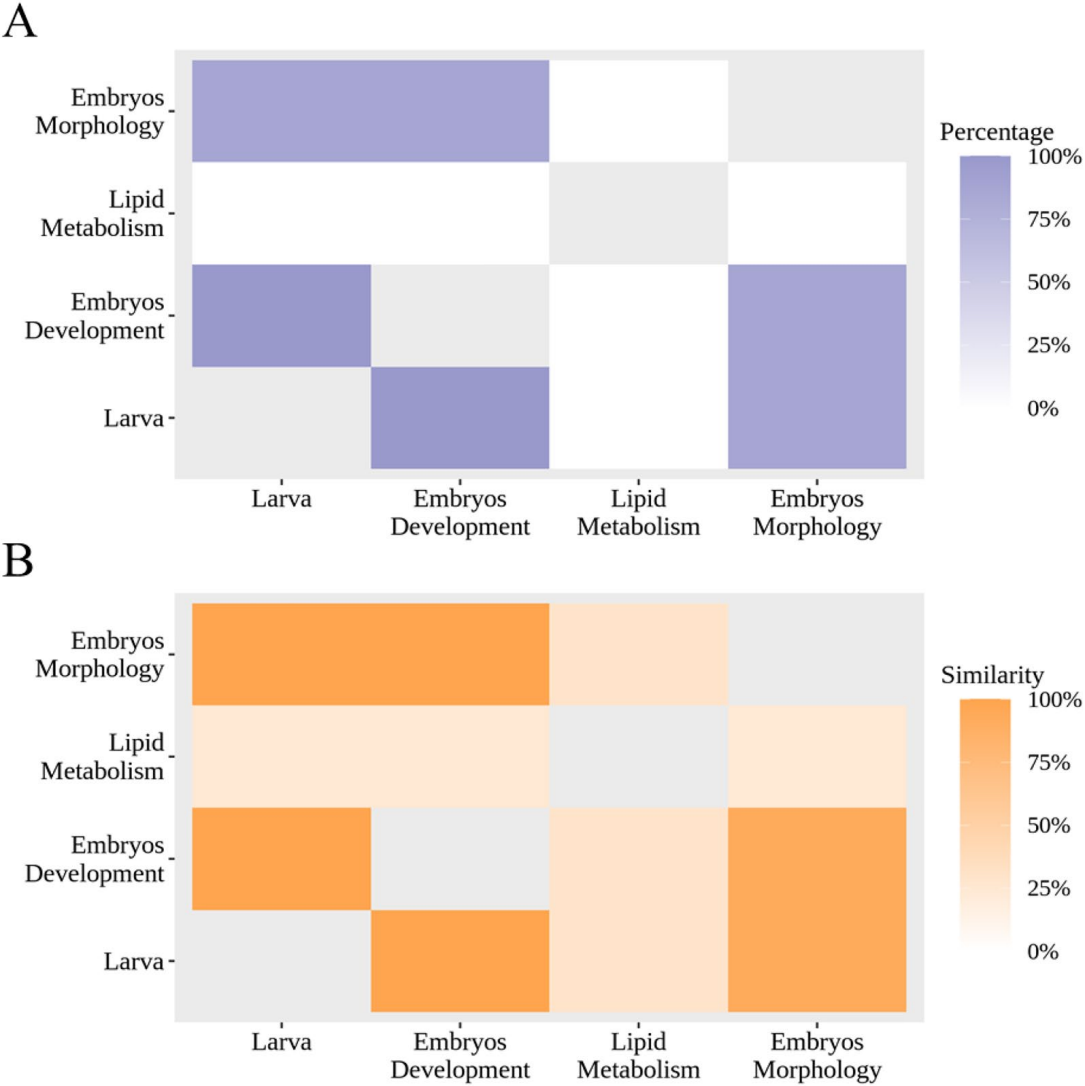
Pairwise comparison of four datasets utilized in this study using **A** the highest percentage of common chemicals and **B** average similarity between datasets

Figure 3 shows the highest percentage of common chemicals and average similarity for each pair of the four datasets. The larva and embryo development datasets were tested against the same chemical list; therefore, the percentage of common chemicals is 100%. The embryo morphology shares 87.1% chemicals with datasets of larva and embryo, and the average similarity is 99.8%. The chemical space of the three datasets is considered highly overlapped. As for the lipid metabolism dataset, only 2 out of the 3801 chemicals were found in the other three datasets giving 0.04% of common chemicals. The average similarities of the lipid metabolism dataset to larva, embryo development, and embryo morphology datasets are 24.6%, 24.6%, and 24.2%, respectively. For the most distinct task of MIC, there are two chemicals in the MIC task associated with a similarity greater than 0.35 with the other tasks. As for the METAB task, 443 chemicals are associated with a similarity greater than 0.35 to larva and embryo development dataset. Those chemicals may contribute to the transfer learning among tasks. Altogether, the lipid metabolism represents a dataset with distinct chemical space compared to the other three datasets. Conventional machine learning algorithms may not be readily applicable to building multitask learning models from datasets with distinct chemical spaces.

**Fig. 4:**
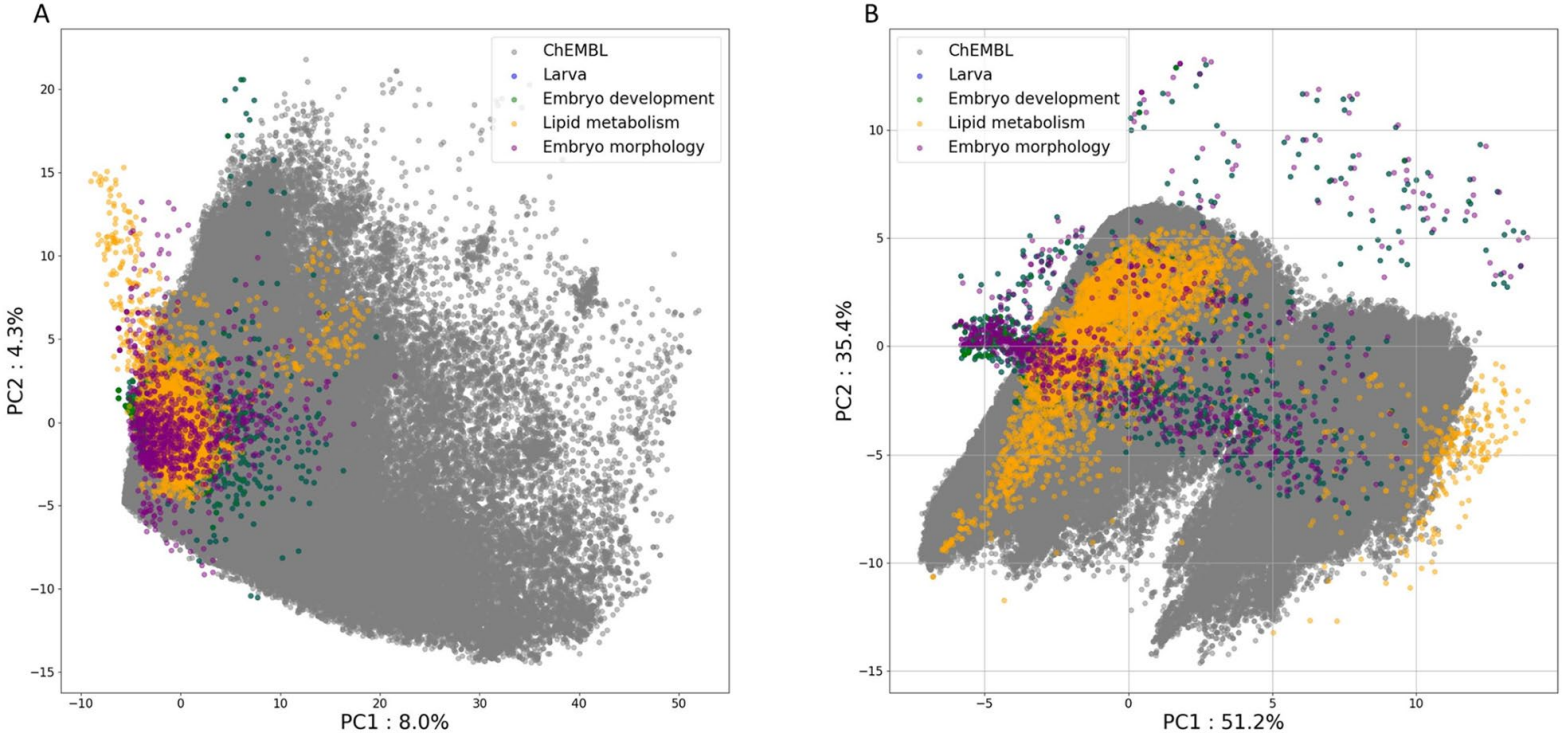
Visualization of chemical spaces of datasets using principal component (PC) analysis based on two fingerprints of **A** ECFP and **B** MACCS

To further investigate the difference of the chemical spaces of the four datasets, principal component analysis was applied to visualize the chemical spaces of the datasets along with the ChEML 34 dataset consisting of 2409,270 compounds [37]. At first, ECFP fingerprint was utilized and the first two principal components were utilized for plotting. As shown in the Fig. 4A, the plot based on ECFP shows some difference between the datasets with a certain degree of overlapped chemical space. However, the variance explained by the first two principal components based on ECFP is only 12.3% indicating insufficient power for showing the distribution of chemicals. To properly capture the variance of the datasets, visualization based on MACCS fingerprint was conducted with a total explained variance of 86.6% using the first two principal components. Figure 4B shows very different chemical spaces occupied by the studied datasets using MACCS-based principal component analysis.

In addition, the absolute correlation coefficients (*r)* of shared chemicals between pairs of the 48 tasks were summarized into four categories of low (|*r*|< 0.3), medium (0.3 ≤|*r*|< 0.5), high (0.5 ≤|*r*|< 0.7), and very high (0.7 ≤|*r*|) consisting of 430, 328, 240, and 37 pairs, respectively. Two tasks of METAB and MIC shared only two or no chemicals with other datasets leading to 93 pairs not categorized. The results indicate that some degree of task similarity existed that could facilitate the knowledge transfer between tasks except for METAB and MIC without sufficient shared chemicals for assessment.

### Multitask learning using MTForestNet

**Fig. 5:**
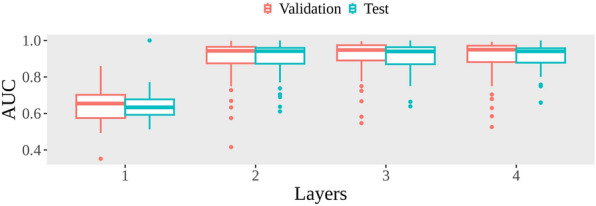
The performance of MTForestNet for each layer

To evaluate the baseline performance of conventional single-task learning algorithms, the popular random forest algorithm was applied to the model development for each of the 48 learning tasks. As shown in Fig. 5, the single-task models (first layer) yielded low average AUCs of 0.642 and 0.638 for validation and independent test, respectively. Considering the complexity of the endpoints, the dataset size may be insufficient for developing robust models with low performance.

To leverage multiple datasets for improving model performance, the MTForestNet was developed for predicting the chemical toxicity of zebrafish toxicity. The proposed framework aimed to improve the prediction by incorporating an additional data dimension of bioactivity obtained from prediction models. The concept is similar to the chemical-biological read-across [38–40] that relevant biological information can facilitate the prediction of endpoints without data. In our experiments with MTForestNet (Fig. 5), the average of validation AUCs for the 48 tasks had a large improvement at the second layer. It converged at the third layer where adding one more layer does not improve the performance. For the validation performance, the MTForestNet achieved a high average AUC of 0.907 with a 26.5% improvement over the single-task models, i.e. the 1st layer of the MTForestNet. As for the test performance, a similar high average AUC value of 0.911 was obtained representing a 27.3% improvement over the single-task models. A total of 43 tasks (89.6%) were well-modeled with AUC values greater than 0.8. Detailed performance is shown in Supplementary Table 2. In contrast, only 1 task with an AUC greater than 0.8 was obtained from single-task models. Please note that the inclusion of the model output from the same task of the previous layer for the model training of the current layer provided 0.3% and 0.7% improvement on AUC over the exclusion of the output. Also, while the convergence rule mentioned in the methodology was utilized to identify the third layer with highest performance, models of the second layer with only slightly worse performance can be useful in resource-limited environments. The proposed MTForestNet multitask learning framework is useful for dealing with multiple datasets with distinct chemical spaces by introducing biological dimensions for improving the prediction of zebrafish toxicity.

**Fig. 6:**
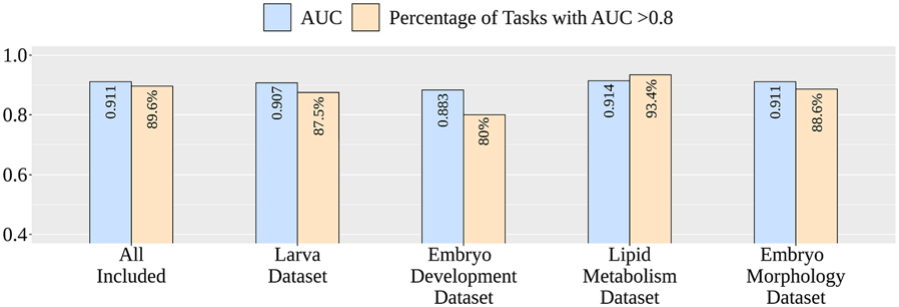
Detailed performance for models trained on different datasets

Since the lipid metabolism dataset is most dissimilar to the other datasets, it is interesting to know whether the exclusion of a single dataset will affect the prediction performance. Four additional experiments of model training, validation, and testing were conducted by excluding one dataset for each experiment. Figure 6 shows the performance comparison of models using all four datasets and only three of them. As expected, the exclusion of the lipid metabolism dataset resulted in a slightly increased average AUC of 0.914 on the test dataset. In contrast, the exclusion of datasets with similar chemical space showed slightly decreased average AUCs of 0.907 and 0.883 for the exclusion of the larva dataset and embryo development dataset, respectively, while the exclusion of the embryo morphology dataset did not affect the average AUC on the test dataset. The analysis of percentages of tasks with an AUC value greater than 0.8 showed the same trend. The percentage of tasks with an AUC value greater than 0.8 for the exclusion of lipid metabolism dataset with dissimilar chemical space was 93.4%, while the worse performance of 87.5%, 80.0%, and 88.6% was obtained for the exclusion of larva dataset, embryo development dataset, and embryo morphology dataset, respectively. The inclusion of the most dissimilar dataset of lipid metabolism only slightly affected the performance. Also, its inclusion largely improved the average AUC on the lipid metabolism dataset from 0.756 to 0.899 with a 14.3% improvement. The analysis showed that the proposed MTForestNet performed well even for the inclusion of datasets with distinct chemical space.

### Comparison to other multitask learning methods

While MTForestNet achieved a high AUC performance in the zebrafish toxicity prediction, it is interesting to compare MTForestNet with existing methods. Three deep neural network-based multitask models from the DeepChem package [13] and an ExtraTrees-based multitask model of MT-ExtraTrees [41] were implemented for comparison. The three multitask (MTL) classification algorithms are ordinary MTL (DC MTL), progressive MTL (DC ProgressiveMTL), and robust MTL (DC RobustMTL). A grid search was performed to identify the parameters giving the highest validation performance for developing the final model with a maximum epoch value of 1000. Two-layer configurations of [200, 100, 50], and [400, 200, 100], as well as three dropout rates of 0.25, 0.15, and 0.1, were considered in this study.

MT-ExtraTrees introduced an additional splitting criterion based on tasks to learn task-specific knowledge and enable multitask learning which is effective in the prediction of chemical properties and toxicity [7, 42]. The best parameter of n_estimators ∈ {1000, 500, 200, and 100} giving the best validation performance was selected for developing the final model for testing. The mtry was set to log2(total feature number) indicating the number of features for building a tree and the probability of evaluating a task-wise split was set to mtry/(total feature number).

**Fig. 7:**
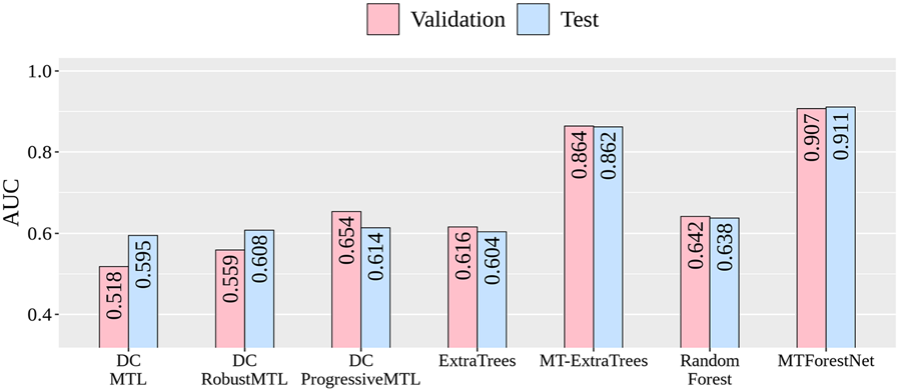
Comparison of MTForestNet and other methods

The comparison of average AUCs for various models is shown in Fig. 7. Please note that single-task models using random forests and ExtraTrees were also implemented for comparison. Detailed performance is shown in Table S2. The proposed MTForestNet capable of handling the datasets with distinct chemical spaces performed best. MT-ExtraTrees with a good AUC of 0.862 on the test sets ranked second, which is 4.9% worse than the MTForestNet. Both multitask methods are superior to their single-task implementations with at least 25% improvement on the average AUC. In terms of the number of well-modeled tasks, there are 43 and 39 tasks with an AUC on the test sets greater than 0.8 for MTForestNet and MT-ExtraTree, respectively.

As for the DeepChem-based models, all the tested models failed to generate good models. The DC_ProgressiveMTL is better than the others with an average AUC of 0.654 on the test sets but is only slightly better than the single-task random forest. The DeepChem-based models may need more parameter tuning to improve the performance. In contrast, MT-ExtraTree and MTForestNet achieved high performance with only a few tuning parameters. In general, MTForestNet performed best in the zebrafish toxicity datasets and held strong potential in addressing the challenge of multitask learning from datasets with distinct chemical spaces.

### Overall toxicity assessment

**Fig. 8:**
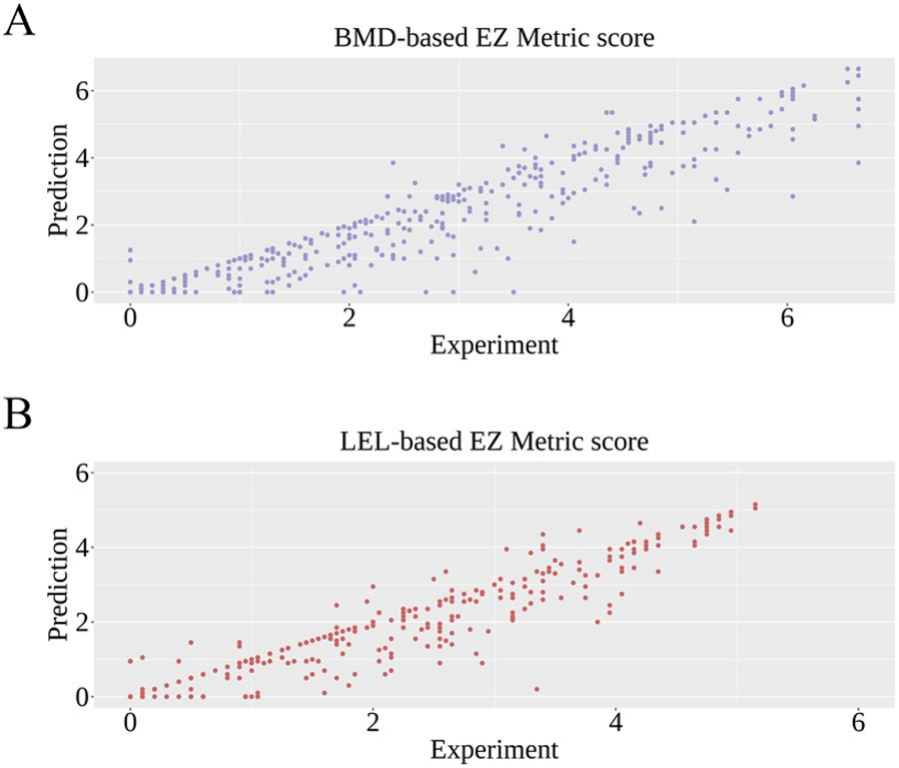
Comparison of predicted and experimental embryonic zebrafish metric (EZ Metric) scores using **A** benchmark dose for 10% effect values (BMD10) and **B** lowest effect level (LEL)

The embryonic zebrafish metric (EZ Metric) is a weighted sum of 21 toxicity endpoints and is useful for representing the overall toxicity in embryonic zebrafish [33]. To assess whether the predicted results can provide good overall toxicity evaluation, the EZ Metric scores based on predicate values and experimental values were calculated and compared as shown in Fig. 8. Based on the collected datasets of larva and embryo development, the EZ Metric scores can be calculated using BMD10 and LEL data, respectively. Please note that the data of three toxicity endpoints in the embryo development data are missing, therefore only data of the remaining 18 toxicity endpoints were utilized for calculating the LEL-based EZ Metric score.

In general, the predicted and experimental EZ Metric scores correlated well with Pearson correlation coefficients of 0.956 and 0.939 for BMD- and LEL-based EZ Metric scores, respectively. For BMD-based estimation, the predicted EZ Metric score tended to conclude higher overall toxicity, while LEL-based estimation tended to give a lower EZ Metric score. For the application of MTForestNet-derived EZ Metric scores, the BMD-based score is expected to be more useful for identifying most of the potentially toxic chemicals for further testing. In contrast, the LEL-based score can be utilized for scenarios requiring a high precision of toxicity prediction. The results demonstrated the use of MTForestNet prediction for quick overall toxicity assessment.

### Predicted Zebrafish toxicities as informative features for improving developmental toxicity prediction

Since the zebrafish toxicity endpoints are important indicators for studying developmental toxicity of chemicals [43]. It is therefore interesting to compare the model performance for predicting developmental toxicity in humans using conventional ECFP-based feature representation and ECFP with predicted zebrafish toxicity values (ZF_features). Two developmental toxicity-relevant datasets of DART and PDT were utilized for evaluating the performance of the combination of structural and complementary ZF_features. For each of the two datasets, one hundred runs of random data partition were conducted to generate 100 training and 100 test sets. For each run, the processes of feature selection, 10-CV, and model development were based on the corresponding training set. An independent test using the corresponding test dataset was conducted to evaluate the test performance of the developed model.

**Fig. 9:**
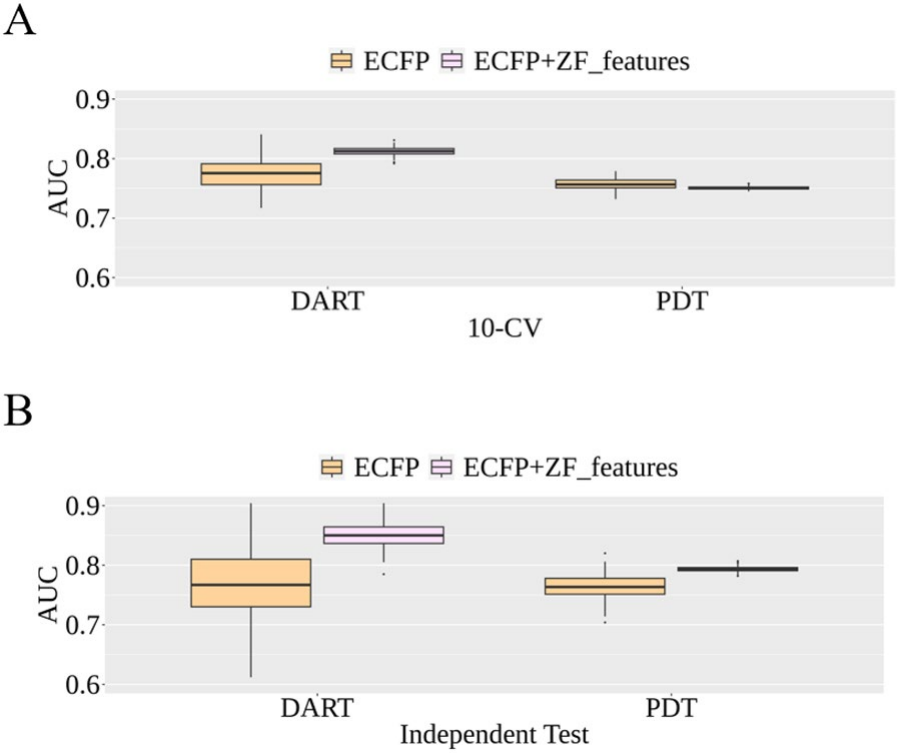
Performance comparison for models using ECFP only and combination of ECFP and ZF_features in **A** tenfold cross-validation (10-CV) using the training sets and **B** independent test using corresponding test sets

The average AUCs of the 100 runs for validation and test are shown in Fig. 9. For the 10-CV performance, the inclusion of ZF_features yielded an average AUC of 0.812 for the DART representing a 3.8% improvement over the conventional ECFP-based model. The average AUC of 0.751 was obtained for the PDT dataset representing a slightly decreased performance (0.6%) compared to the ECFP-based model. In addition to the average AUC, the variance of 100 runs was also an important indicator of the robustness of the models. Figure 9A shows that the inclusion of ZF_features provides a more robust 10-CV performance in both datasets with a much smaller variance in 100 runs compared to ECFP-based models. The independent test results (Fig. 9B) showed that the incorporation of ZF_features provided 8.1% and 4.3% improvement over ECFP-based models with average AUC values of 0.851 and 0.794 for the DART and PDT tasks, respectively. Similarly, a much smaller variance was obtained by incorporating ZF_features in both datasets showing its robustness.

Table S4 compiles the most frequently selected ZF_features based on the 100 runs for the DART and PDT tasks. The common ZF_features among the top 10 features selected for DART and PDT tasks were SM24, LEL_SWIM, and LEL_MORT representing the BMD10 for the absence of spontaneous movement (24 hpf), LEL for the failure of the swim bladder to inflate (120 hpf) and LEL for cumulative mortality by 120 h post fertilization (hpf), respectively. The utilization of ECFP along with complimentary ZF_features led to a notable AUC enhancement over the conventional ECFP-based models for the two developmental toxicity-relevant datasets.

## Conclusion

The sparsity of chemical toxicity data is a common issue in developing robust models. This study proposed a novel multitask learning method MTForestNet for leveraging multiple datasets with distinct chemical spaces. The MTForestNet is a progressive learning algorithm based on random forest classifiers organized in a neural network-like multilayer architecture. A total of 48 datasets of zebrafish toxicity were collected and utilized for benchmarking the proposed MTForestNet. The experimental results showed that MTForestNet exhibited a high AUC performance of 0.911 in the test sets. It provided superior performance over the compared single-task and multitask learning algorithms. In addition, the developed models can be reliably utilized to assess the overall chemical toxicity of embryonic zebrafish based on the EZ Metric scoring method with a high correlation coefficient greater than 0.9 to the experimental results. The ZF_feature generated from the developed models can be utilized in combination with conventional ECFP features to improve the performance and robustness for predicting developmental toxicity. The models are expected to be useful for zebrafish toxicity studies. Since chemical toxicity and bioactivity data are usually sparse and associated with distinct chemical spaces, the proposed MTForestNet method can be potentially useful for learning from the chemical toxicity and bioactivity data.

### Supplementary Information

Supplementary material 1.

## Data Availability

Code and datasets are available at https://github.com/m946107011/MTForestNet.git.

## Abbreviations

AUC: Area under the receiver operating characteristic curve
BMD10: Benchmark dose for 10% effect
DART: Developmental and Reproductive Toxicity
DC: DeepChem
ECFP: Extended connectivity fingerprints
EZ Metric: Embryonic zebrafish metric
GCN: Graph convolutional neural network
Hpf: Hours post-fertilization
LEL: Lowest effect level
LEL_MORT: LEL for cumulative mortality by 120 h post fertilization
LEL_SWIM: LEL for failure of swim bladder to inflate by 120 h post fertilization
MIC: Minimum inhibition concentration
MTL: Multitask
PDT: Prenatal developmental toxicity
SM24: The BMD10 for absence of spontaneous movement by 24 h post fertilization
TOX_SCO: Developmental toxicity score
Tox21: Toxicology in the 21st century
ZF_features: Zebrafish toxicity values
10-CV: 10-Fold cross-validation
