# Supplementary material for: A novel multitask learning algorithm for tasks with distinct chemical space: zebrafish toxicity prediction as an example
Source: J Cheminform. 2024 Aug 2;16:91. doi: 10.1186/s13321-024-00891-4 (PMC11297603; doi:10.1186/s13321-024-00891-4)
Supplement: Supplementary file 1 — Supplementary material 1. [file 13321_2024_891_MOESM1_ESM.docx]

# Supporting Information

Table S1. Task description.

| Dataset | Tasks | Description | Toxic/nontoxic chemicals |
| --- | --- | --- | --- |
| Larva [[15]](https://www.zotero.org/google-docs/?XTNlVw) | MO24 | Mortality before 24 hours post fertilization (hpf) | 18.5% |
|  | DP24 | Delayed development (24 hpf) | 19.0% |
|  | SM24 | Absence of spontaneous movement (24 hpf) | 2.9% |
|  | NC24 | Notochord malformation (wavy notochord) (24 hpf) | 3.1% |
|  | MORT | Cumulative mortality (120 hpf) | 26.3% |
|  | YSE | Yolk sac edema, swelling around the yolk sac (120 hpf) | 22.1% |
|  | AXIS | Curved or bent axis in either direction (120 hpf) | 20.0% |
|  | EYE | Eyes malformation, missing or smaller/larger than normal (120 hpf) | 17.2% |
|  | SNOU | Snout shortened or malformation (120 hpf) | 20.7% |
|  | JAW | Jaw malformation (120 hpf) | 20.4% |
|  | OTIC | Otic Malformation or missing (120 hpf) | 12.1% |
|  | PE | Heart malformation, pericardial edema (fluid around the heart) (120 hpf) | 20.4% |
|  | BRAI | Brain malformation or necrosis (120 hpf) | 16.7% |
|  | SOMI | Somites malformation or disorganization, missing (120 hpf) | 12.0% |
|  | PFIN | Pectoral fin malformation or missing (120 hpf) | 13.1% |
|  | CFIN | Caudal fin malformation or missing (120 hpf) | 14.7% |
|  | PIG | Lack of pigmentation, overpigmentation (120 hpf) | 12.4% |
|  | CIRC | No blood circulation or flow (120 hpf) | 10.0% |
|  | TRUN | Short trunk, malformation or missing (120 hpf) | 15.6% |
|  | SWIM | Failure of swim bladder to inflate (120 hpf) | 11.2% |
|  | NC | Notochord malformation (wavy notochord) (120 hpf) | 8.8% |
|  | TR | Not responsive to touch (120 hpf) | 17.4% |
|  | MOV21 | Change in movement at light-to-dark transition time point  (MOV21 measures the immediate change in fish movement when transitioning from light to dark by calculating the difference in movement values at 9 and 10 minutes (MOV2 - MOV1).) | 55.0% |
|  | AUC21 | Change in area under movement versus time curve between dark and light periods  (AUC21 quantifies the cumulative change in the area under the curve of fish movement during dark and light periods, with AUC1 covering the 3 to 9-minute period in light conditions and AUC2 spanning the 10 to 16-minute period in darkness) | 52.1% |
| Embryo development (Truong, Reif, et al. 2014) | LEL_MORT | Cumulative mortality by 120 hours post fertilization (hpf) | 42.0% |
|  | LEL_YSE | Yolk sac edema, swelling around the yolk sac (120 hpf) | 19.0% |
|  | LEL_AXIS | Curved or bent axis in either direction (120 hpf) | 15.5% |
|  | LEL_EYE | Eyes malformation, missing or smaller/larger than normal (120 hpf) | 11.3% |
|  | LEL_SNOU | Snout malformation or shortened (120 hpf) | 16.3% |
|  | LEL_JAW | Jaw malformation (120 hpf) | 16.3% |
|  | LEL_OTIC | Otic malformation or missing (120 hpf) | 6.1% |
|  | LEL_PE | Heart malformation, pericardial edema (fluid around the heart) (120 hpf) | 17.3% |
|  | LEL_BRAI | Brain malformation or necrosis (120 hpf) | 8.0% |
|  | LEL_SOMI | Somites malformation or disorganization, missing (120 hpf) | 5.2% |
|  | LEL_PFIN | Pectoral fin malformation or missing (120 hpf) | 7.9% |
|  | LEL_CFIN | Caudal fin malformation or missing (120 hpf) | 7.6% |
|  | LEL_PIG | Lack of pigmentation, over pigmentation (120 hpf) | 6.0% |
|  | LEL_CIRC | No blood circulation or flow (120 hpf) | 2.7% |
|  | LEL_TRUN | Short trunk, malformation or missing (120 hpf) | 8.6% |
|  | LEL_SWIM | Failure of swim bladder to inflate (120 hpf) | 4.4% |
|  | LEL_NC | Notochord malformation (wavy notochord) (120 hpf) | 1.7% |
|  | LEL_TR | Not responsive to touch (120 hpf) | 10.9% |
| Lipid metabolism [[13]](https://www.zotero.org/google-docs/?KmyixK) | METAB | Each well was scored visually based on the fluorescence observed in the gall bladder and intestine of each fish.  (5-day post-fertilization larvae were placed in 96-well plates and exposed to test compounds overnight at a concentration of 25 uM in a 2% DMSO solution. Subsequently, the larvae were stained with PED-6 for 6 hours.) | 1.4% |
|  | MIC | Lowest concentration that gave either complete or partial inhibition of fluorescence. | 24.0% |
| Embryo morphology [[12, 14, 16]](https://www.zotero.org/google-docs/?ERMLdp) | 18_END_LEC | Zebrafish LEC based on Min. LEC of 18 endpoints (120 hpf) | 54.4% |
|  | MOR_LEC | Zebrafish LEC for mortality (120 hpf) | 41.1% |
|  | SUBLETH_17_END_LEC | Zebrafish LEC based on Min. LEC of 17 sublethal endpoints (120 hpf) | 28.2% |
|  | TOX_SCO | In order to formalize the descriptive data (lethality and hatching status) with the numerical data (Malformation Index), the author assigned the descriptive data a numerical score: 40 for lethality and 20 for non-hatching, and if the larva was alive and hatched, then the Toxicity Score was equal to the Malformation Index. (120 hpf) | 74.0% |

Table S2. Comparison with other multitask methods.

| Methods | Validation | | | | Test | | | | # Task (AUC > 0.8) |
| --- | --- | --- | --- | --- | --- | --- | --- | --- | --- |
|  | AUC | ACC | SENS | SPEC | AUC | ACC | SENS | SPEC |  |
| MTForestNet | 0.907 | 89.8% | 51.1% | 93.2% | 0.911 | 89.1% | 44.9% | 92.8% | 43 |
| Random Forest | 0.642 | 83.3% | 9.8% | 92.5% | 0.638 | 83.3% | 9.7% | 92.7% | 1 |
| MT-ExtraTrees | 0.864 | 84.5% | 63.3% | 88.0% | 0.862 | 83.4% | 63.3% | 85.9% | 39 |
| ExtraTrees | 0.616 | 82.3% | 38.9% | 82.3% | 0.604 | 82.1% | 33.2% | 82.7% | 1 |
| DC MTL | 0.518 | 81.5% | 17.0% | 83.3% | 0.595 | 80.7% | 25.0% | 82.1% | 0 |
| DC ProgressiveMTL | 0.654 | 89.3% | 9.6% | 91.7% | 0.614 | 89.5% | 8.2% | 91.7% | 2 |
| DC RobustMTL | 0.559 | 83.9% | 15.9% | 85.8% | 0.608 | 83.9% | 22.9% | 85.3% | 2 |

Table S3. The weights of individual toxicity endpoints.

| Endpoints | Weight |
| --- | --- |
| MO24 (24 hpf) | 1 |
| DP24 (24 hpf) | 0.3 |
| SM24 (24 hpf) | 0.2 |
| NC24 (24 hpf) | 0.4 |
| MORT (120 hpf) | 0.95 |
| YSE (120 hpf) | 0.5 |
| AXIS (120 hpf) | 0.4 |
| EYE (120 hpf) | 0.2 |
| SNOU (120 hpf) | 0.1 |
| JAW (120 hpf) | 0.2 |
| OTIC (120 hpf) | 0.1 |
| PE (120 hpf) | 0.6 |
| BRAI (120 hpf) | 0.6 |
| SOMI (120 hpf) | 0.1 |
| PFIN (120 hpf) | 0.1 |
| CFIN (120 hpf) | 0.1 |
| PIG (120 hpf) | 0.1 |
| CIRC (120 hpf) | 0.2 |
| TRUN (120 hpf) | 0.3 |
| SWIM (120 hpf) | 0.1 |
| TR (120 hpf) | 0.1 |

Table S4. Top 10 ZF_features selected for the DART and PDT datasets.

| DART | | PDT | |
| --- | --- | --- | --- |
| Endpoints | Frequency | Endpoints | Frequency |
| **SM24** | 54 | EYE | 81 |
| BRAI | 53 | LEL_CFIN | 69 |
| TOX_SCO | 53 | 18_END_LEC | 61 |
| **LEL_SWIM** | 46 | **SM24** | 52 |
| **LEL_MORT** | 25 | **LEL_SWIM** | 41 |
| OTIC | 24 | LEL_TR | 31 |
| LEL_AXIS | 18 | MOV21 | 30 |
| AXIS | 16 | **LEL_MORT** | 27 |
| LEL_CIRC | 15 | LEL_EYE | 27 |
| LEL_NC | 14 | MO24 | 23 |
